# What are the support experiences and needs of patients who have received bariatric surgery?

**DOI:** 10.1111/hex.12423

**Published:** 2015-11-02

**Authors:** Melanie Sharman, Martin Hensher, Stephen Wilkinson, Danielle Williams, Andrew Palmer, Alison Venn, Douglas Ezzy

**Affiliations:** ^1^ Menzies Institute for Medical ResearchUniversity of TasmaniaHobartTasmaniaAustralia; ^2^ Department of Health and Human ServicesHobartTasmaniaAustralia; ^3^ Royal Hobart HospitalHobartTasmaniaAustralia; ^4^ School of NursingUniversity of TasmaniaHobartTasmaniaAustralia; ^5^ School of Social SciencesUniversity of TasmaniaHobartTasmaniaAustralia

**Keywords:** bariatric surgery, health policy, health services, obesity, obesity surgery

## Abstract

**Objective:**

To explore the support needs and experiences of patients who had received publicly or privately funded bariatric surgery and the importance of this support in mediating outcomes of surgery.

**Methods:**

Seven semi‐structured focus groups were conducted. A broad interview schedule guided the discussions which were audio‐recorded and transcribed verbatim. Data were analysed thematically.

**Results:**

Twenty‐six women and 15 men with a mean age of 54 years (range 24–72) participated in the study. Participants described support needs from health professionals, significant others (family and friends), peers (bariatric surgery recipients) and the general community. Peer, dietetic and psychological support were identified as important factors influencing the outcomes (e.g. weight reduction or health improvement) or experience of bariatric surgery but were identified as infrequently received or inadequately provided. Psychological support was proposed as one of the most significant but commonly overlooked components of care. Support needs appeared higher in the first year post‐surgery, when subsequent related or unrelated surgeries were required and following significant life change such as worsening health. For some participants, deficits in support appeared to negatively influence the experience or outcomes of surgery.

**Conclusion:**

Providers of bariatric surgery should discuss support needs and accessibility regularly with patients especially in the first year post‐surgery and following significant change in a patient's life (e.g. declined health or childbirth). Nutrition, psychological and peer support (e.g. through support groups) may be especially important for some patients.

## Introduction

The provision of bariatric surgery for the treatment of obesity and related comorbidity has been increasing across both developed and developing countries.^1^ Bariatric surgery is generally recommended for those with intractable class 2 obesity [body mass index (BMI) 35–39.9 kg/m^2^] and obesity‐related comorbidity (e.g. type 2 diabetes mellitus) or class 3 obesity (BMI ≥ 40 kg/m^2^) with or without obesity‐related comorbidity.^2^, ^3^, ^4^ Although long‐term outcomes from randomized controlled trials are lacking, long‐term observational studies infer more durable weight loss, improved health status and reduced mortality risk following bariatric surgery compared with non‐surgical interventions.^5^, ^6^, ^7^ The most common bariatric surgery types internationally are sleeve gastrectomy, gastric bypass and laparoscopic adjustable gastric banding (LAGB).^8^

Recommendations for multidisciplinary or multimodal support pre‐ and post‐bariatric surgery are often provided.^2^, ^3^, ^4^, ^9^, ^10^, ^11^ However, while results from qualitative and quantitative studies on bariatric surgery may imply or directly point to various support needs of bariatric surgery recipients (e.g. addressing disordered eating behaviours, deficits in nutritional status or challenges in adjusting to post‐surgical life^12^, ^13^, ^14^, ^15^), high‐quality research is lacking on the type of support and its characteristics that are valued by recipients of bariatric surgery and how support affects surgical outcomes.^3^, ^11^, ^16^ Consequently, this study sought to investigate the support experience and needs of publicly or privately funded LAGB recipients and the impact of support on surgical outcomes. To our knowledge, a study of this type has not been conducted previously. This information is important for health service planning and delivery.

## Methods

This study was conducted in Tasmania, an island state of Australia, which has a population of just over 500 000 with approximately 35 000 adults estimated to have class 2 obesity or greater.^17^ There are two public hospitals and three private hospitals that conduct publicly and privately funded bariatric surgery (principally LAGB), respectively. Over 4500 LAGB surgeries occurred in Tasmania between July 2003 and July 2013, the highest rate per capita in Australia.^18^

A multidisciplinary model of care is recommended across most Australian jurisdictions providing publicly funded bariatric surgery.^19^, ^20^, ^21^ Patients receiving publicly funded LAGB in Tasmania, principally access additional health professional support through the private sector if they can afford to, otherwise limited support is available through the public system. Like elsewhere, websites of private bariatric surgery clinics in Tasmania indicate that other health professionals (e.g. nutritionists or dietitians) are either on staff or may be required in patient care. Generally reference to other support types (e.g. support groups) appears lacking.

### Design

Data were collected through semi‐structured focus groups. Significantly, more females than males have bariatric surgery in the general population despite comparable levels of obesity and most bariatric surgery are privately funded in Australia.^22^, ^23^ For these reasons, the groups were same‐sexed and separated by surgery funding type to enable greater exploration of differences due to these characteristics. Ethics approval was granted by the University of Tasmania's Health and Medical Human Research Ethics Committee.

### Recruitment

The study was advertised in three local newspapers, through radio and within related departments at the Royal Hobart Hospital, Tasmania. Letters were sent to private LAGB patients (*n* = 180) by one author (SW, bariatric surgeon) and by the Department of Health and Human Services (through author MH) to public LAGB patients (*n* = 127) using a stratified and randomized approach. Additionally, SW provided interested and eligible patients with the study's information sheet. To ensure confidentiality, identifying details of participants were not shared between investigators.

### Procedure

At the point of enquiry, prospective participants were provided with an overview of the study and asked several demographic and clinical screening questions. This information was used to determine the general characteristics of those responding to the recruitment methods and to inform subsequent purposive sampling. Where numbers permitted participants were selectively invited to attend a focus group ensuring a mix of demographic and clinical characteristics.

Prior to engaging in the focus group, invited participants were provided with the study's information sheet and informed consent was obtained. To enhance consistency, one of the authors (MS) assisted or led all focus groups. The duration of each focus group was no longer than 1.5 h. The discussion schedule focused on the following: the types of support received or desired; the impact or anticipated impact of the various types of support discussed; the barriers to receiving support; and how the surgical outcomes could be improved. The schedule was informed by a review of the literature and consultation with public health experts, policymakers, primary and tertiary health professionals, experienced qualitative and quantitative researchers and people with experience of obesity. Of the seven focus groups conducted, five were held in Hobart (the largest city in Tasmania, with a hospital operating at the highest teaching and referral level) and two in Launceston (a smaller regional city with an accredited teaching hospital).

### Data analysis

All focus groups were audio‐recorded and transcribed verbatim. The transcripts were checked and de‐identified. The thematic analysis was descriptive and interpretive and facilitated by use of software (NVivo 10; QSR International, Doncaster, Victoria, Australia). The emerging themes were discussed with, and reviewed by, other investigators also familiar with the transcripts. In total, the transcripts were re‐read several times. All quotes cited below are from participants.

### Quality control

An audit trail was kept for the project that included transcripts, question schedules, memos, notes on research team meetings, a project logbook and reflective notes.

## Results

One hundred and sixteen adults over 18 years old who had received publicly or privately funded bariatric surgery expressed interest in being involved in the study, of which 41 participated in the focus groups Seven focus groups were conducted between August and October 2014 which included three focus groups for females (mean age 53.3 years, range 31–72) and two for males (mean age 59.2 years, range 47–69) who had received privately funded LAGB, and one focus group for females (mean age 48.2 years, range 24–66) and two for males (mean age 58.3 years, range 41–66) who had received publicly funded LAGB. One focus group for males included those who had received either publicly or privately funded LAGB. Additional focus groups were not conducted because data saturation had been achieved. A summary of clinical and demographic characteristics of participants is provided in Table 1.

**Table 1:** Participant characteristics (*n* = 41)

Characteristic	
Age	
Mean (range)	54 (24–72)
Gender	
Female (%)	63
Education	
Completed year 12 or less (%)	42
Current BMI^a^	
Mean (range)	35 (21–62)
Maximum BMI^a^	
Mean (range)	47 (32–69)
Surgery funding type	
Private (%)	78
Time since surgery (years)	
Mean (Range)	6 (0–31)

a Self‐reported.

Although overweight and obesity are different measures, the participants indicated a preference for the term ‘overweight’ when describing their bodies, which is consistent with the literature.^24^ Therefore, despite differences in BMI between participants, the term overweight was used in the focus groups.

While there is some evidence of sex differences in the experience of bariatric surgery,^25^ clear differences in support needs between the sexes were not evident in our data. Men and women consistently discussed a similar range of support needs and experiences. Meaningful comparisons between surgery funding type could not be made due to the low number of participants who had received publicly funded bariatric surgery.

The main categories of support needs identified by participants were from health professionals, peers (recipients of bariatric surgery), significant others (family and close friends) and the general community.

### Health professional support experiences and needs

#### Dietary support

Diet‐related support experiences and needs were discussed extensively across all focus groups. A few participants talked about the benefits of receiving professional dietetic input in that it provided important reinforcement or new know‐ledge about the surgery or that it facilitated or helped maintain behaviour change post‐surgery. For example, one participant said:

Once she [the dietitian] seen me I started to lose weight, and she also gave me some advice on how to manage the eating with the lap‐band. (male, public surgery)

Some participants said that not receiving comprehensive dietetic support was unfavourable:

I mean I did see a dietitian for a short period before I had the surgery, but I had no advice on what or how to eat post‐operatively and I really missed that. I can see it would have been most beneficial to have had that support. (male, public surgery)

A couple of participants said they could not access or continue to receive professional dietary support because it was too expensive. For a few participants, the dietitian they had consulted lacked knowledge about the needs of bariatric surgery recipients. One participant described how this impacted them:

And it's only now that I've got a dietitian that's actually done work in the field [bariatric surgery], that she's seeing me, that I'm starting to come out the other end. (female, private surgery)

Others said that advice received did not always suit their circumstances (e.g. recommending unaffordable foods). In two focus groups, it was proposed that outside of professional dietetic input, providing recipes or conducting cooking demonstrations would be helpful dietary supports.

Participants described several situations in which eating and drinking could be challenging, indicating additional areas where support may be needed. For example, many talked about the necessity to reduce food and liquid intake for a few days following a gastric band adjustment and the difficulties with eating out. Others discussed that maintaining adequate hydration was challenging particularly when cold drinks were not tolerated. Some experienced fainting, constipation or headaches that they attributed to inadequate fluid intake. Participants also talked about day‐to‐day variance in food tolerance suggesting difficulties in achieving a balanced diet:

It's not logical; some days you can eat something and the next day you can't [Many agree]. One day you can drink a lot and the next day you can't. But it's not logical, you can't quite figure it out. (female, private surgery)

Several participants expressed or demonstrated shortfalls in nutrition literacy. For example, a male and female spoke about their experience of iron deficiency. Another participant said it was several years after surgery before she could eat fruit and vegetables.

A summary of the key diet‐related support themes and additional quotes is included in Table 2.

**Table 2:** Key dietary support themes and examples of related verbatim quotes

Themes	Quotes
1. Benefits of professional dietary support	‘I've got half‐an‐hour with her [the dietitian] to discuss things and that's made the world of difference’ (female, private surgery)
2. Impact of not receiving professional dietary support	‘So you really don't have that knowledge from a dietitian about what sort of foods are going to be good, how to maintain your nutrition, how to maintain the nourishment you need for your body. What's all this protein stuff about that you keep hearing all the time?’ (female, private surgery)
3. Shortfalls in dietary advice received	‘she [dietitian] wanted me to eat all these fancy things and I thought, “I can't afford half this stuff” (female, public surgery)
	‘she [the dietitian] was telling me to eat things that clearly I couldn't eat you know’ (male, private surgery)
4. Dietary challenges	‘So if I'm sitting in a restaurant or somebody's dining room table I have to be very careful how much I eat, otherwise I just got to excuse myself from the table and go to the bathroom and have a spit’ (male, public surgery)
	‘you know you can't eat or drink as much – particularly say ah two to three days after the adjustment and then it sort of settles down’ (male, public surgery)
5. Shortfalls in nutrition literacy	‘…and then I got very, very, very ill from not eating enough nutritious food’ (female, private surgery)

#### Psychological support

Psychological support was considered by some as one of the most important but frequently overlooked components of pre‐ and post‐bariatric surgery care and suggested as even more important than professional dietetic support. Yet, few had received psychological support and of those who spoke about seeing a psychologist the experience had been favourable:

Because you do get depressed when you're overweight. There's no shame – you know I was clinically depressed. I have now started to see a psychologist which is helping. (female, private surgery)

There were many examples in the group discussions that signalled the potential role for psychological support. One participant stated that to some extent, they had substituted alcohol for food as a means to receive the ‘comfort’ (female, private surgery) that food had previously provided. In all focus groups, participants talked extensively about the psychological aspects of becoming or being overweight. Many shared their histories of complex relationships with food (e.g. ‘food addiction’, female, private surgery) and disordered eating behaviour – characteristics that were not always changed by surgery even when weight had been lost:

Mindset's massive. I cried for a month when I had my lap‐band because I couldn't eat. And I was depressed, like full on depressed because I couldn't eat what I wanted to eat cause I'm addicted to food… And I didn't expect that reaction to not being able to eat food. That was amazing for me, a real eye‐opener. And then in a month I was like ‘I really am addicted to food. I really have a problem’ (female, private surgery)

Further, adapting to the lap‐band was described by one participant as ‘tough’ (male, private surgery) and there were frequent accounts of stressful events to manage, such as dealing with food blockages or eating out. Social habits were often modified because of limitations in what could be eaten or fearing that others would recognize that they had a LAGB because of changed eating habits. Social challenges seemed more apparent among those reluctant to broadly disclose their history of bariatric surgery.

Additionally, some participants spoke about their expectations of surgery and the disappointment experienced when these were not realized. Males and females talked about being dissatisfied with having excess skin. A few participants had experienced body dysmorphia – seeing their current body as its pre‐surgery size even when significant weight had been lost: ‘A lot of the time it doesn't matter what weight you actually are, you're fat still in your mind’ (female, private surgery). Others talked about their reliance on the lap‐band stating that they lacked ‘self‐control’ (male, public surgery) or did not trust themselves in its absence – perspectives which, for some, were reinforced when weight was rapidly regained following prolonged band deflation for clinical or personal reasons.

And I would never want it [the lap band] to come out either. Because I don't think I trust myself. Because of that mental thing that's never been addressed or whatever, if I had it out I reckon I'd just put all the weight back on again. That's what scares the shit out of me. (female, private surgery)

A summary of the key psychological support themes and additional quotes is included in Table 3.

**Table 3:** Key psychological support themes and examples of related verbatim quotes

Themes	Quotes
1. Experience of professional psychological support	‘I see the psychologist, it's just because I started to feel different. I sort of thought my friends were looking at me different. Whether I'm – I'm not a threat to them or anything because they're all tiny and skinny I was just starting to feel I was a bit isolated now more than before. But anyway that's me and I'm dealing with that – because you do feel lots of different feelings’ (female, private surgery)
2. Indications for psychological support	‘…I'm still battling – my mindset never went away with the operation’ (female, private surgery)
	‘And how much of a trauma it is that you've actually got to really retrain yourself in thinking, “I'm not hungry,” because most of us have been binge eaters, and comfort eaters, and whatever. There is just so little support out there’. (female, private surgery)
	‘There is a tough stuff about it, there's nothing easy about it [New speaker: That's right (male, public surgery)]. It drags at ya and you've got to work at it. And you know mentally you know you've got to toughen up on it’. (male, private surgery)
	‘…and if I go out to dinner somewhere and I'm a bit stressed, I have to go to the toilet. I've not been out to dinner where I haven't had to go and throw up what I've eaten because I get stressed and conscious of people – they're probably not watching me, but I'm really conscious of what they're thinking’. (female, private surgery)
	‘Mine [excess skin] hangs down quite low – I can actually get there and [New speaker: Yeah, yeah so can I.] lift it up. And it's frustrating and you sort of feel embarrassed about it’. (female, public surgery)
	‘I was inspired by a much younger girl – that had lost all her weight through having a lap‐band. And I'm really disappointed in myself because I haven't done the same thing’. (female, private surgery)

#### Support from the bariatric surgeon

Generally, the discussion across the groups indicated a desire for comprehensive and accessible information about the surgery that included the longer term. The discussion also indicated a need for information to be provided through a mix of electronic and non‐electronic media to cater for individual learning styles and Internet access or use. Although some participants felt they had received sufficient information, others felt there were gaps, for example in respect to excess skin, details of the procedure, necessary dietary change, complications and exercising guidelines.

It would have been good to get more support when you needed knowledge – if you could ring somebody and have a line. (male, private surgery)

A number of participants appeared to have limited knowledge of the type or potential benefits of available supports and some suggested it was the role of the surgeon to provide this information and initiate referral as needed. Others who viewed the LAGB as a tool rather than a cure appeared to take more responsibility for initiating their own additional care or seeking information, characteristics that seemed to contribute to better outcomes. For example, one participant who viewed the surgery as successful said: ‘I make myself go back regularly [to the surgeon] because I've had problems with mine and make sure that I'm being as proactive as you're allowed to be’ (female, private surgery).

A few participants had not seen the surgeon for a long time (e.g. several years) even when surgery‐related difficulties (e.g. complications) had been experienced. For some of these participants, it appeared that more regular follow‐up with the surgeon could have been helpful, for example those experiencing enduring dietary challenges or complications. One participant who had less weight loss success than some others said:

I've never been back to the surgeon since, and that was about six years ago; and I won't. And maybe – it's just not right not being able to eat all the good things and still being able to eat crap, you know and that's what you're sustained on; rubbish. (female, private surgery)

Further, although some participants had experienced significant surgery‐related difficulties (e.g. complications), these experiences did not necessarily adversely influence how they felt about having a LAGB. This could be linked to the attitude conveyed by some that the surgery was their last opportunity to reduce their weight or improve their health, suggesting that they were prepared to endure difficulties in order to realize this goal.

#### General practitioner support

In all focus groups, there were participants who spoke about the benefits of regularly consulting with their general practitioner (GP), particularly among those who had an extended history with the same GP. The benefits discussed included: having a single point of contact who had knowledge of their full health profile, regular monitoring of vitamin and mineral levels and general health, providing support about the decision to have LAGB; being a source of information about bariatric surgery, and providing support to engage in healthy lifestyle behaviours.

My GP's fantastic. She continues to monitor all my vitamin levels, etcetera – more regularly than I even want to think about, because she's scared of me getting ill again, and you know she's been brilliant. (female, private surgery)

However, not all participants felt that they were supported by their GP in their decision to have a LAGB, although two participants said that their GP's opinion changed when they lost weight.

### Social support experiences and needs

#### Peer support (recipients of bariatric surgery)

Many participants spoke about the potential or realized benefits of having access to people who had received bariatric surgery to share experiences and information, both before and after their own surgery. In two focus groups, it was suggested that having access to a bariatric surgery recipient who had a relevant professional background or training would be useful. For some, the preferred medium for peer support was face‐to‐face, and others had found social media (e.g. Facebook) to be an important and influential source of support:

So there's a lot of support there for you know people that have access and can do what they can on Facebook. (male, private surgery)

I've found social media to be a huge help for me [New speaker: Yes]. I mean there's the Tassie lap‐band one, there's a Aussie lap‐band one, and there's even the American ones [New speaker: I like the Tassie one]… but it's been great. It's answered a lot of questions that no one else has answered, or no one else had brought up [New speaker: Yes, I've found that]. (female, private surgery)

One participant suggested that social media provided a means to discuss related personal issues that people may feel disinclined to discuss with their doctor. Others acknowledged that the information available through social media was not always welcomed: ‘But you've got to take it [social media] for what it is, and you've got to be strong enough to take what you need from it’ (female, private surgery). The general absence of a moderator was also discussed as a potential problem. However, not all participants were aware of the presence or extent of social media dedicated to recipients of bariatric surgery.

Most of the focus groups ended with participants discussing how beneficial it had been coming together and sharing their experiences and some expressed a desire to connect again.

#### Significant others (family and friends)

There was variety within and across the groups regarding the extent to which participants had told significant others about having bariatric surgery and the support received from those who knew of their surgery. For some, there was reluctance to tell others because of negative views about the merits of bariatric surgery (e.g. it was the easy way out) or because of experiencing emotions such as embarrassment. Others talked about significant others (e.g. partners) initially being unsupportive but later changing their position when ‘success’ had been experienced. Some, but not all, participants, experi‐enced negative consequences from not disclosing or severely limiting disclosure of their bariatric surgery to others [e.g. feeling ‘depressed’ (female, private surgery)], although some felt that the alternative was also unfavourable (e.g. fear of judgement).

Many felt that support from significant others was essential: ‘I couldn't do it without my family’ (female, private surgery). However, there were also examples of significant others lacking consideration of the consequences of the surgery: ‘…with my in‐laws, if we go there they insist on making a sandwich. I just can't eat bread’ (female, private surgery). A few participants discussed the benefits of significant others also being recipients of bariatric surgery. For example, one participant said she had benefited greatly from her daughter's prior experience with a LAGB – ‘I couldn't cope without my daughter’ (female, private surgery).

#### The general community

Many participants talked about the impact and extent of weight and bariatric surgery‐related stigma and discrimination experienced in the general community, including within the workplace, health and media sectors. One participant felt she had to continually prove that she ‘wasn't the lazy that goes with the fat’ (female, private surgery). Others felt that they were treated as if they were ‘moronic’ (female, public surgery) because of their overweight bodies and another said that she felt more respected by others after having lost weight following surgery. Some participants talked about a perception in the general community that having bariatric surgery ‘was the coward's way out’ (male, private surgery) and how incorrect this attitude was because life with a LAGB can be ‘tough’, ‘there's’ nothing easy about it’ (male, private surgery). There was also discussion about the lack of community understanding about how difficult it is to lose weight and maintain the loss as evidenced by receipt of comments such as ‘I don't know why anyone would want to have that operation it's not worth the money, it's not worth anything. If they just ate right, if they just exercise right they wouldn't need the operation’ (male, private surgery).

However, others talked positively about the support they had received from their extended communities including within the workplace. One participant discussed the reaction from work colleagues when he told them he had a LAGB: ‘Well good on you, it's good to see that you've finally had something done to fix some of the problems’ (male, public surgery). Another felt it was unnecessary to tell work colleagues because she considered their support unimportant. One participant spoke about how supportive people had been in the town where she lived and how it had inspired her ‘not to bail’ (female, private surgery) and to try her best to make the LAGB work for her.

### Critical periods for support

Discussion across the groups indicated support needs were likely to be higher in the first year following surgery when learning to adapt to the requisite behaviour change. One participant said she had ‘never been more fragile’ in the first year after surgery. Another participant outlined why he thought the first year was so challenging:

You know the truth's the truth, let's face it – every big person loves food, eats fast, and eats plenty of it you know. You know so we've had lap‐band surgery and we've adjusted it up so now the psychology of it where we can't eat fast, we can't eat a lot of it and that's probably the most difficult part over the [first] 12 months. (male, public surgery)

Further, there were several vulnerable times for weight regain discussed: if band deflation was needed for personal or clinical reasons (e.g. subsequent surgeries); when there was an enduring decline in health (e.g. because of injury or medication prescribed for comorbidity) or following childbirth.

…the reason I'm 10 kilos over my goal weight at the moment is I had the lap‐band relaxed so I could go to that [special event]… and I use the device as a crutch. I don't actually have a lot of self‐control. (male, public surgery)

And then I was in a car accident last year and … I put on weight again because I couldn't do any exercises at all. (female, public surgery)

## Discussion

The numerous, diverse, individualized and on‐going support needs described by participants highlight the complex experiences of obesity and its surgical treatment. The chronic nature of obesity also implies that bariatric surgery may be distinct from other types of elective surgery. Further, the need for comprehensive support and its impact on some participants is unsurprising given that: the suppression of hunger through bariatric surgery addresses one aspect of the often multifaceted issue of overweight^2^; significant sustained behaviour change unique to the procedure is needed following surgery; and patients may experience surgery‐related complications.^26^, ^27^

Psychological and dietary needs were discussed extensively across the focus groups, although few participants had received assistance from psychologists or dietitians, which may have influenced surgical outcomes. Guidelines on bariatric surgery commonly recommend a multidisciplinary approach which includes specialist psychological/behavioural and dietetic intervention,^2^, ^3^, ^4^, ^9^, ^10^, ^11^ but dietitians and psy‐chologists are not uniformly incorporated into bariatric surgery models of care.^16^, ^28^ Further, there are gaps in the evidence on how best to provide these services and their effect.^3^, ^11^, ^28^

The Australian guidelines described on‐going roles for primary health‐care professionals in the management of bariatric surgery recipients that reflect some of those valued by participants in this study.^2^ For example, regular monitoring of general health and providing support to engage in healthy lifestyle behaviours.^2^ Consequently, encouraging patients to regularly consult with their GP is recommended.

The literature commonly espouses the need for on‐going support following bariatric surgery.^2^, ^4^, ^9^ In our study, some participants who reported lack of follow‐up, particularly with the bariatric surgeon, did less well than others and reported difficulties with adjusting to the LAGB or enduring complications. Further, some participants appeared to minimize the impact of surgery‐related difficulties which may be attributed to a strong desire for the surgery to be successful. This finding is a signal to health professionals that more careful discussion about the well‐being of their patients may be necessary. Further, we found that participants who viewed the LAGB as a tool rather than a cure tended to talk more about their own role in the success of the LAGB and they seemed more likely to report better outcomes. This implies that patients may do better if they have realistic expectations of the outcomes of surgery.

The group discussions indicated a desire for comprehensive and accessible information about the surgery (including into the longer term) provided through both electronic and non‐electronic media. Patients who have received or are planning bariatric surgery commonly use the internet to source related information,^29^, ^30^, ^31^ consequently this is an important medium for health professionals to use to communicate with their patients. Guidance on components of patient education is available 11 although high‐quality studies in this area are lacking.

A systematic review of 10 observational studies determined that social support (support groups or support by significant others) experienced post‐bariatric surgery was positively correlated with weight loss.^32^ The American 2013 clinical guidelines on the perioperative support of bariatric surgery recipients^10^ recommended that all patients be encouraged to attend social support groups into the long‐term (Grade B level evidence). Participants in this study spoke strongly about the positive or potential influence of face‐to‐face or social media peer support pre‐ and post‐surgery. Electronic peer support has the advantage of being generally accessible although evidence on how best to provide this support still appears to be lacking subsequent to a 2004 systematic review.^33^ Taken together, the promotion or inclusion of social support as part of a patient's care plan should be considered.

A 2009 review by Puhl and Heuer^34^ highlighted the pervasive nature of weight‐based stigmatization and discrimination across multiple sectors in society (e.g. health, education, the workplace) and the negative psychological and physiological consequences that it may induce (e.g. depression and disordered eating). Participants in this study had experienced weight‐based stigma and discrimination including that related to having received bariatric surgery – a finding recently reported in the literature.^35^ Health professionals are encouraged to consider how their practice may be perpetuating weight‐based stigma and discrimination and the consequences of this.

Finally, we have identified that there may be periods post‐surgery where support needs are higher, for example in the first year following surgery or following significant change in circumstances such as declining health. Consequently, health professionals are alerted to the potential fluctuations in support needs of patients over the life of the intervention.

Our suggestions on how the outcomes of bariatric surgery may be improved through non‐surgical means can be found in Box 1.

Box 1 **Our suggestions on how the outcomes of bariatric surgery may be improved through non‐surgical means**

- Provide comprehensive and accessible information to patients through a combination of electronic and non‐electronic media
- Regularly check patient understanding of key messages particularly those that are dietary related
- Regularly discuss support needs and experiences with patients
- Evaluate the psychological needs of patients often
- Where indicated, refer patients to allied health professionals who have knowledge of bariatric surgery. If financial constraints are present, refer patients to relevant and reputable sources of information or programs that are low cost or free
- Encourage patients to regularly consult with their GP
- Minimize loss to follow‐up where possible
- Promote the availability and potential role of support groups
- Check for the presence of weight or bariatric surgery related stigma or discrimination in clinical practice and remediate as necessary
- Fill training gaps related to bariatric surgery in health professional education through university curricula and professional development.

## Conclusion

This study has highlighted that patients’ experience of support may influence the outcomes of bariatric surgery and that quantitative investigation of this relationship is warranted. Health professionals are encouraged to involve patients in determining their support needs and design consultations that ensure gaps in support are identified and remediated. Whilst it is acknowledged that some providers of bariatric surgery may include comprehensive and multidisciplinary support, the findings of this study provide useful prompts for checking models of care. Where financial constraints exist providers of bariatric surgery are encouraged to direct patients to free or low cost support options as necessary and appropriate.

## Sources of funding

This work was supported by an Australian National Health and Medical Research Council (NHMRC) Partnership Project Grant (APP1076899). AV is supported by a NHMRC Research Fellowship.

## Conflict of interests

None to declare.
